# Simultaneous profiling of the blood and gut T and B cell repertoires in Crohn’s disease and symptomatic controls illustrates tissue-specific alterations in the immune repertoire of individuals with Crohn’s disease

**DOI:** 10.3389/fimmu.2025.1638522

**Published:** 2025-09-05

**Authors:** Aya K. H. Mahdy, Valentina Schöpfel, Gert Huppertz-Hauss, Gøri Perminow, Florian Tran, Corinna Bang, Johannes R. Hov, May-Bente Bengtson, Petr Ricanek, Randi Opheim, Tone Bergene Aabrekk, Trond Espen Detlie, Vendel A. Kristensen, Mathilde Poyet, Marte Lie Høivik, Andre Franke, Hesham ElAbd

**Affiliations:** ^1^ Institute of Clinical Molecular Biology, Kiel University and University Hospital Schleswig-Holstein, Kiel, Germany; ^2^ Department of Gastroenterology, Telemark Hospital, Skien, Norway; ^3^ Department of Pediatrics, Oslo University Hospital, Oslo, Norway; ^4^ Norwegian PSC Research Center, Department of Transplantation Medicine, Division of Surgery, Inflammatory Diseases and Transplantation, Oslo University Hospital, Oslo, Norway; ^5^ Institute of Clinical Medicine, Faculty of Medicine, University of Oslo, Oslo, Norway; ^6^ Research Institute of Internal Medicine, Division of Surgery and Specialized Medicine, Oslo University Hospital, Oslo, Norway; ^7^ Section of Gastroenterology, Department of Transplantation Medicine, Division of Surgery and Specialized Medicine, Oslo University Hospital, Oslo, Norway; ^8^ Department of Gastroenterology, Vestfold Hospital Trust, Tonsberg, Norway; ^9^ Department of Gastroenterology, Lovisenberg Diaconal Hospital, Oslo, Norway; ^10^ Department of Gastroenterology, Oslo University Hospital, Oslo, Norway; ^11^ Department of Public Health, Institute of Health and Society, University of Oslo, Oslo, Norway; ^12^ Medical Department, Vestfold Hospital Trust, Tonsberg, Norway; ^13^ Department of Gastroenterology, Akershus University Hospital, Lørenskog, Norway; ^14^ Institute of Experimental Medicine, Kiel University, Kiel, Germany; ^15^ Institute for Digestive Research, Lithuanian University of Health Sciences, Kaunas, Lithuania

**Keywords:** IBD - inflammatory bowel disease, T cell repertoire, B cell repertoire, immune repertoire analysis, colon immune repertoire, blood immune repertoire

## Abstract

**Introduction:**

Crohn’s disease (CD) is a clinical subset of inflammatory bowel disease that is characterized by patchy transmural inflammation across the gastrointestinal tract. Although the exact etiology remains unknown, recent findings suggest that it is a complex multifactorial disease with contributions from the host genetics and environmental factors such as the microbiome. We have previously shown that the T cell repertoire of individuals with CD harbors a group of highly expanded T cells which hints toward an antigen-mediated pathology.

**Methods:**

We simultaneously profiled the αβ and γδ T cell repertoire in addition to the B cell repertoire of both the blood and the colonic mucosa of 27 treatment-naïve individuals with CD and 27 age-matched symptomatic controls.

**Results:**

Regardless of disease status, we observed multiple physiological differences between the immune repertoire of blood and colonic mucosa. Additionally, by comparing the repertoire of individuals with CD relative to controls, we observed different alterations that were only detected in the blood or colonic mucosa. These include a depletion of mucosal-associated invariant T (MAIT) cells and an expansion of *TRAV29/DV5-TRAJ5* ^+^ clonotypes in the blood repertoire of individuals with CD. Also, a significant depletion of multiple *IGHV3-33-IGHJ4^+^* and *IGHV3-33-IGHJ6^+^* clonotypes in the blood and gut IGH repertoire of individuals with CD.

**Discussion:**

Our findings highlight the importance of studying the immune repertoire in a tissue-specific manner and the need to profile the T and B cell immune repertoire of gut tissues as not all disease-induced alterations will be detected in the blood.

## Introduction

Inflammatory bowel disease (IBD) is a chronic, inflammatory condition of the gut that is clinically subclassified into two main entities, Crohn’s disease (CD) and ulcerative colitis (UC). CD is characterized by severe, transmural inflammation that is observed in patches across the entire gastrointestinal tract. In contrast, UC is observed in the colon and rectum with a continuous superficial inflammation that is restricted to the mucosa and the submucosal layer. Dysregulated immune responses have been shown to be a central theme in CD, for example, we have recently shown that yeast specific CD4^+^ T cells exhibit a more cytotoxic phenotype in CD (1). Furthermore, we have also identified a group of type II like natural killer T (type II NKT) cells that we referred to as Crohn’s associated invariant T (CAIT) cells which are more expanded in individuals with CD relative to healthy controls and individuals with UC (2).

A key determinant of the antigenic specificity of a T cell is its T cell receptor (TCR), which is generated by a somatic recombination process known as V(D)J in which multiple gene segments encoding the different chains of a TCR recombine to generate a diverse repertoire of TCRs. In humans, four TCR chains are utilized to form TCRs, namely, α, β, γ, and δ TCR chains. Generally, α and β chains dimerize to form TCRs observed in αβ T cells, and γ and δ chains dimerize to form TCRs observed in γδ T cells. Based on the class of antigens they recognize; T cells can be classified into conventional T cells, which recognize peptides presented by the human leukocyte antigen (HLA) proteins, and unconventional T cells which recognize a wider set of non-peptide antigens. Conventional T cells are αβ T cells that recognize peptides presented by HLA-I and HLA-II proteins. Despite this, there is a wide spectrum of unconventional αβ T cells such as mucosal-associated invariant T cells (MAIT) which recognize vitamin B metabolites presented by MHC-related protein 1 (MR1) (3). Conversely, γδ T cells are generally unconventional, recognizing a versatile set of non-peptide antigens such as phosphoantigens presented by butyrophilins (4), sulphatide presented by CD1d (5), and different stress markers (6).

Similar to T cells, B cell receptors (BCR) are the main determinant of the antigenic specificity of B cells. BCRs are also generated by V(D)J recombination which forms three main antigen-binding chains, namely, Ig heavy chain (IGH) and two Ig light chains, κ (IGK) and λ (IGL). In addition, the heavy chain can have one of five main constant regions: μ, γ, α, δ and ϵ which are used in Immunoglobulin M (IgM), G (IgG), A (IgA), D (IgD), and E (IgE) antibodies, respectively. Although BCRs and TCRs are generated by a similar process, *i.e.* V(D)J recombination, and have considerable similarities, BCRs differ in three main features. First, BCR recognizes their antigens directly without any need for antigen presentation by a presenting molecule such as HLA, CD1d, or MR1. While some γδ T cell subsets can recognize their antigens directly without a presenting molecule (7), most T cells are restricted by their presenting molecule. Second, due to mechanisms such as somatic hypermutation, the genetic sequence of BCRs keeps changing after the formation of functional BCR and antigen stimulation. On the other hand, once T cells egress from the thymus their TCRs are fixed and will not change. Lastly, BCR produces antibodies that have a much higher affinity to their antigenic target in comparison to TCRs.

The repertoire of BCRs, which is a collection of unique BCRs in a sample, can be profiled by sequencing the V(D)J recombination products encoding for these receptors, through B cell receptor repertoire sequencing (BCR-Seq). Using this framework, Bashford-Rogers and colleagues (8), profiled the B cell repertoire of six different autoimmune diseases showing an increase in the clonality of IgA in CD and systemic lupus erythematosus, additionally, the authors showed a skewing in the frequency of different IGHV genes in these different diseases. However, these results were obtained using blood samples which might not recapitulate the local immune repertoire of the gut. Indeed, the correlation between the B cell repertoire in the gut and the blood remains understudied, for example, Weisel and colleagues (9) recently identified a new subset of gut specific B cells. These results highlight the importance of profiling the local repertoire of affected tissues. In this study, we profiled the αβ and γδ T cell repertoires, as well as the B cell repertoire of paired blood and gut samples (obtained using biopsies) of 27 treatment-naïve individuals with CD and 27 age-matched symptomatic controls (total =108 samples) using an amplification strategy the enables the T and B cell repertoire to be simultaneously profiled in the same library preparation reaction. We started our analysis by analyzing the overlap between the blood and the gut repertoires independent of the disease status, enabling physiological differences between the blood and the gut repertoire to be investigated. After that we analyzed the repertoire of each anatomical compartment individually in order to discover disease-induced changes in the blood as well as the colon.

## Material and methods

### Sample collection

Blood and colon biopsies were collected during the diagnostic work-up of participants included in the IBSEN III study. Individuals referred to the hospitals under the suspicion of IBD were invited. Patients who fulfilled the Lennard Jones criteria (10) for CD were included while individuals referred with suspicion of IBD but where diagnostic work-up, including endoscopy and histology, was completely normal were included as symptomatic controls. Two mucosal biopsies were collected from the left colon using jumbo biopsy forceps and immersed in Allprotect ^©^ (Qiagen) before incubation at 2-8°C overnight and then freezing at -80°C. Blood was drawn in 6 ml EDTA tubes and frozen at -80°C.

### DNA extraction

DNA extraction was performed using *Chemagic STAR* (*Hamilton*) with the *chemagic*™ *DNA Blood 10k Kit H12* from *Revvity* for isolating high-quality DNA. DNA was isolated from biopsies using a *QIAcube Connect* device and the *DNeasy Blood and Tissue Kit* (Qiagen). Quality control (QC) for the extracted DNA was performed using Agilent TapeStation Systems. The quality of extracted DNA ranged from 6.5-9.7 (DIN score). DNA concentration measurements were performed by Invitrogen™ Qubit™ dsDNA-BR (Broad Range) assay kit with qubit fluorometer, such as Victor Nivo multimode microplate reader, (PerkinElmer).

### Library preparation, sample pooling, sequencing, and clonotype identification

The 7genes DNA multiplexing kits from MiLaboratories were used for profiling the full repertoire of T and B cells from the gut and blood samples (108 samples prepared from 56 different individuals). 120ng of DNA were used for library preparation according to the manufacturer’s instructions. Subsequently, these libraries were pooled together and cleaned with magnetic beads (AMPure, ^®^XP Beads protocol, Beckman Coulter, with 1:0.8 sample: beads ratio). Lastly, samples were diluted to a final concentration of ~2nM and 60 µl of this pool were then submitted for sequencing at the Competence Centre for Genomic Analysis (CCGA) Kiel. Approximately 10% of PhiX was added to the pool which contained 118 samples (108 samples from 56 individuals, 8 negative controls, and 2 positive controls) prior to sequencing on a single S4 lane on a NovaSeq 6000 machine using a paired 150bp x2 approach.

From these 118 samples, 2,742,957,859 reads were generated with a median of 21,215,996.5 and a mean of 23,245,405.58 reads per samples. Across all samples, a median of ~ 45.65% and a mean of ~48% of reads could not be aligned by MiXCR (11) using the “milab-human-dna-xcr-7genes-multiplex” preset, consequently these reads were not used for clonotype identification. Furthermore, the aligned reads did not have a uniform distribution across all loci with a median of ~10.9% of reads aligning to the TRA, ~3.43% to the TRB, ~52.2% to the TRG, ~11.55% to the TRD, ~4.93% to the IGH, ~5.37% to the IGK and ~2.28% to the IGL loci. The quality and quantity of the input DNA were a fundamental challenge for profiling the immune repertoire from mucosal biopsies where the utilization of low-quality DNA usually fail to produce trustworthy immune profiles. For all the analyses reported here, we removed the immune repertoire of two samples because of their initial low quality input DNA.

### Clonal expansion index

The clonal expansion index can be interpreted as the count of the most expanded clonotype over the total count of all clonotypes, that is the fraction of sequencing reads belonging to the most expanded clonotype relative to the sum of all clonotypes’ reads. Mathematically it is defined as follow:


$$Clonal\;expansion\;index=\frac{\max\left(C\right)}{\sum_{i\in C}C_{i}}$$

Where max(*C*) represents the maximum number of reads a clonotype has over all the clonotypes defined in the set *C* of clonotypes. Consequently, $\sum_{i\in C}C_{i}$ represents the sum of reads identified in the clonotype set *C*.

## Results

### Cohort description

**Figure 1:**
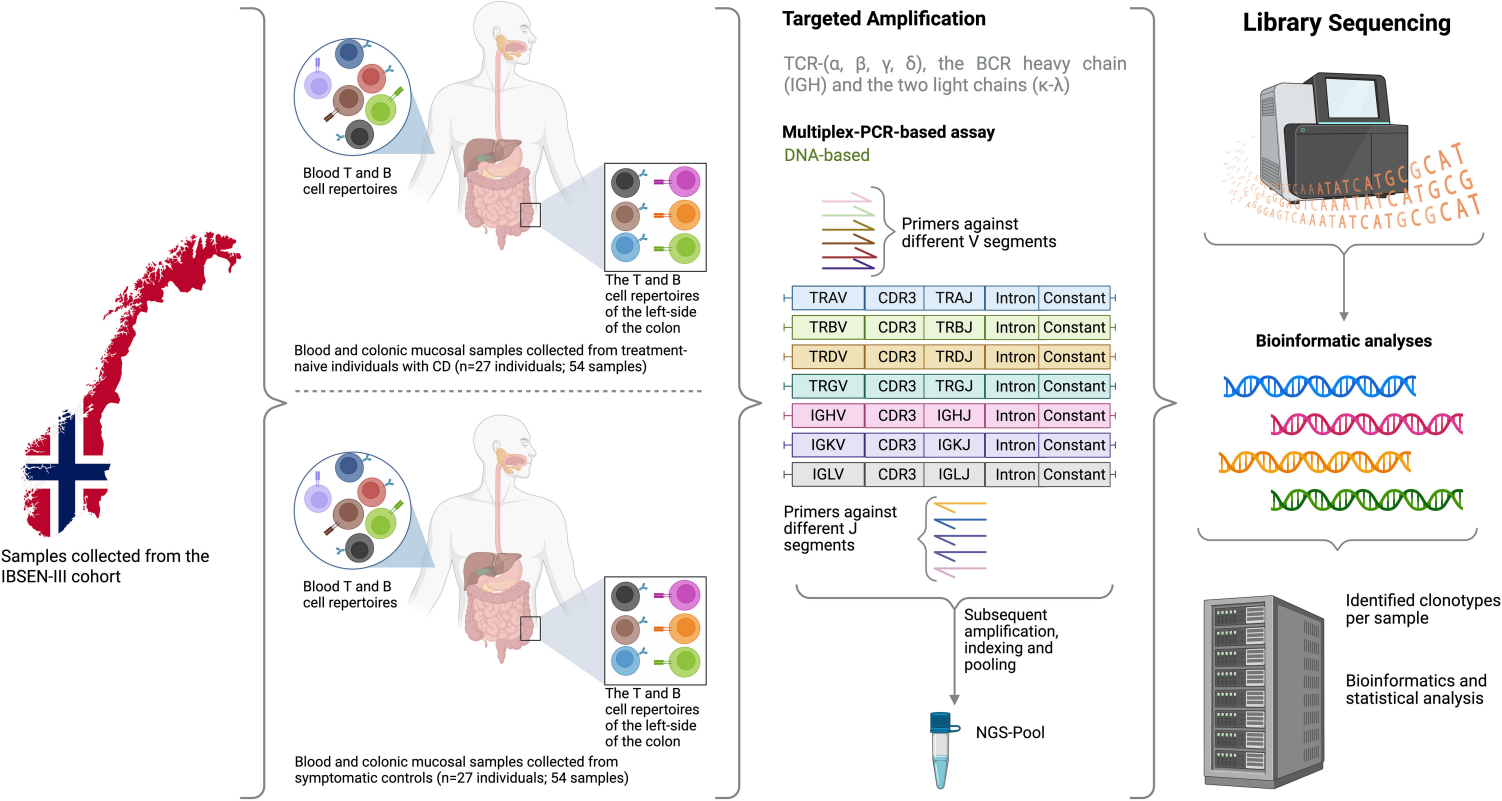
An overview of the study design where samples from 27 treatment-naïve individuals with CD and 27 sex and age-matched symptomatic controlled from the Norwegian IBSEN-III cohort were included. For each individual, DNA was extracted from peripheral blood and the left-side of the colon, after that, the DNA was used to amplify the T cell repertoire including TCR-(α, β, γ, δ), the BCR heavy chain and the two light chains (κ-λ). After library preparation and the addition of a unique index to each library, samples were pooled together and sequencing using 150bpX2 sequencing. Lastly, bioinformatic analyses were used to reconstruct the repertoire of each library from the generated sequencing reads and several statistical analyses were used to compare the repertoire of blood and gut as well as investigate the difference in the repertoire of individuals with CD relative to symptomatic controls. Created in BioRender. ElAbd, H. (2025) https://BioRender.com/rc1ut5d.

We used samples collected from the inception-cohort inflammatory bowel disease in South-Eastern Norway III (IBSEN-III) (12). Specifically, we used DNA extracted from EDTA blood tubes and biopsies derived from colonic mucosa (gut biopsies hereafter) collected at the time of diagnostic endoscopic procedure to profile the full T and B cell repertoire (Material and Methods; **Figure 1**) of 27 active (inception; recently diagnosed) treatment-naïve individuals with CD and 27 age-matched symptomatic controls (*i.e.* individuals referred with suspicion of IBD but whose diagnostic work-up, including endoscopy and histology, was normal) (**Table 1**). For the 27 controls, biopsies were derived from non-inflamed tissues from the left-side of the colon, while for individuals with CD, we utilized 11 biopsies from inflamed and 16 from non-inflamed colons (left-side). Following the immune profiling we aimed at investigating two questions, first, the physiological differences in the immune repertoire of these two tissues, second, similarities and differences in CD-induced immune changes that are either detected at the local gut immune repertoire and/or at the blood repertoire level.

**Table 1:** Phenotypic and clinical parameters of individuals with CD and symptomatic controls included in the study.

	Crohn’s disease (n=27)	Symptomatic controls (n=27)
Age	27.07 ± 6.13 (18-39)	27.81± 6.28 (19-38)
Female percentage	44% (n=12)	59% (n=16)
Disease location	L1 (Ileum): 6 individuals L2 (Colon): 7 individuals L3 (Ileocolon): 14 individuals L4 (Upper GI tract): 2^†^ individuals	N/A
Behavior overtime	B1 (NSNP^#^): 16 individuals B2 (Stricturing): 7 individuals B3 (Penetrating): 2 individuals	N/A

^†^The two individuals with upper GI tract disease location also had disease observed at the ileocolon, i.e. they were L3+L4 cases. ^#^Represents cases with non-structuring, non-penetrating (NSNP) disease.

### Different immune receptor loci exhibit a different degree of overlap between the blood and the gut, and show different degrees of interindividual overlap

**Figure 2:**
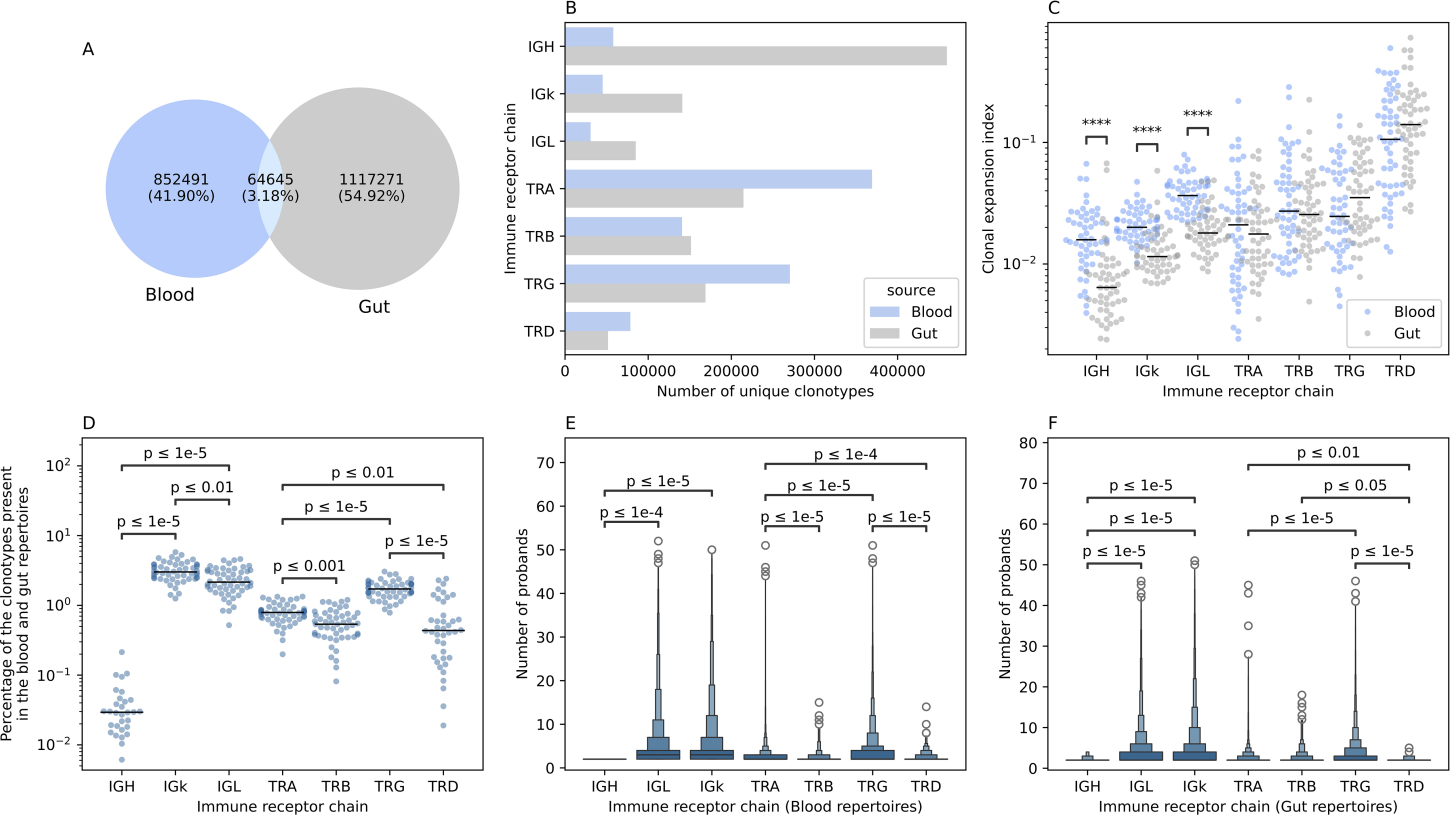
The overlap between the blood and the gut repertoires among the seven different immune receptor chains. **(A)** Overlap in productive clonotypes between the blood and the gut repertoires among all immune receptor loci. **(B)** The number of clonotypes observed by each immune receptor chain in the gut and the blood. **(C)** Clonal expansion index of clonotypes observed in the blood and the gut repertoire of different immune receptor chains. **(D)** Percentage of overlapped clonotypes between clonotypes detected in the blood and the gut repertoires over the union of clonotypes observed in the gut and the blood repertoires of the different chains. **(E, F)** boxen plot representation of the degree of publicity, i.e. the number of probands having the same clonotype in their repertoire, of public blood and public gut clonotypes, respectively. In **(C-F)**, statistical comparisons were conducted using the two-sided Mann-Whitney-Wilcoxon test with Bonferroni correction for multiple testing. In this figure and all subsequent figures unless stated otherwise, the asterisks are used to represent the P-values, with n.s. donating non-significant (P>0.05), *represents P ≤ 0.05, **donates, P ≤ 0.01, ***means P ≤ 0.001 and lastly, ****designates a P ≤ 0.0001.

Across all samples and profiled immune receptor chains, namely, TRA, TRB, TRG, TRD, IGH, IGK, and IGL, 2,034,407 unique productive clonotypes, *i.e.* unique V(D)J arrangement that did not contain a frameshift or a stop codon, were identified. Among them, 852,491 were observed only in blood samples (~42%) while 1,117,271 (~55%) were identified from gut tissues and only 64,645 (~3%) were detected in the repertoire of both compartments (**Figure 2A**). For two donors, (one CD and one control) we observed a small number of clonotypes which also correlated with a low-quality input DNA, subsequently, samples derived from these two individuals were excluded from all analyses. From an immune receptor chain perspective, there was a clear separation between the immune repertoire of gut tissues and the blood with a more diverse T cell repertoire in blood and a more diverse B cell repertoire in the gut (**Figure 2B**). Specifically, the TRA and the TRG showed higher diversity in the blood compared to gut tissues, while the IGH showed substantially greater diversity in the gut than in the blood (**Figure 2B**). To explore this further, we calculated the clonal expansion index (Methods) of each locus in both blood and gut samples (**Figure 2C**). We did not observe any significant difference in the clonal expansion index of the T cell repertoires of gut and blood, across the four loci (**Figure 2C**). However, there was a significant difference in the clonal expansion index of the B cell repertoires (IGH, IGK, IGL), with the blood repertoires showing a higher degree of clonal expansion compared to the local gut B cell repertoires (**Figure 2C**).

We next quantified the overlap between the blood and colon repertoires at the intra-individual level as well as the overlap between the blood and gut repertoires across different individuals (inter-individual level variation). Among the B cell repertoire chains, the IGK showed the highest degree of intra-individual overlap between the blood and the gut immune repertoires (median ~3%). In contrast, the IGH repertoire the lowest overlap between these two compartments (median ~0.03%) (**Figure 2D**). The IGL showed an intermediate level of overlap between the blood and gut (median ~2.15%) which is significantly lower than the IGK but nearly 71 times higher than the IGH repertoire (**Figure 2D**). A similar pattern was observed for the T cell repertoire chains. The TRG showed the highest degree of overlap (median ~1.68%), followed by the TRA loci (median ~0.78%) (**Figure 2D**). The least overlap was observed for the TRB and the TRD loci, with a median of ~0.53% and ~0.43%, respectively (**Figure 2D**).

A similar pattern was observed at the inter-individual level for the blood repertoire, where the light chains of the BCR, *i.e.* IGL, and IGK, are shared among a higher number of individuals, with a median of three individuals, relative to the heavy chain, *i.e.* IGH, with a median of two (**Figure 2E**). It is worth noticing that this analysis focused on public clonotypes which, by definition, are clonotypes observed in two or more individuals. At the T cell repertoire level, the TRG repertoire was the most public among the four TCR chains with a mean of ~3.5 individuals, followed by the TRA (mean ~2.56 individuals), then the TRD (~2.35 individuals) and lastly the TRB (mean ~2.29 individuals) (**Figure 2E**). Across the four chains the median was 2, suggesting that these differences are driven by sub-populations of highly public clonotypes, that are present in many individuals. These populations were more frequent in the TRG repertoire followed by the TRA, the TRD and lastly, the TRB repertoire. A similar pattern was observed at the gut immune repertoire with the light chains being more public relative to the heavy chain, however, IGK clonotypes were slightly more public than IGL clonotypes with a mean of 3.97 and 3.74, respectively (**Figure 2F**). At the T cell repertoire level, there was not any significant difference in the degree of publicity between the TRA and the TRB repertoires, with the TRG repertoires exhibiting the highest degree of publicity relative to all other TCR chains (**Figure 2F**). The term degree of publicity refers to the number of individuals where a specific clonotypes was observed, for example, a clonotype with a degree of publicity of five, means that it was identified in five individuals. Hence, it represents the degree of sharing a clonotypes in the population. Public clonotypes are more amenable to statistical analyses, for example, to analyze their human leukocyte antigen (HLA) restriction (13, 14) and their association with diseases and prior infections (15–19).

### The blood and gut immune repertoire significantly differ in the utilized V and J genes

**Figure 3:**
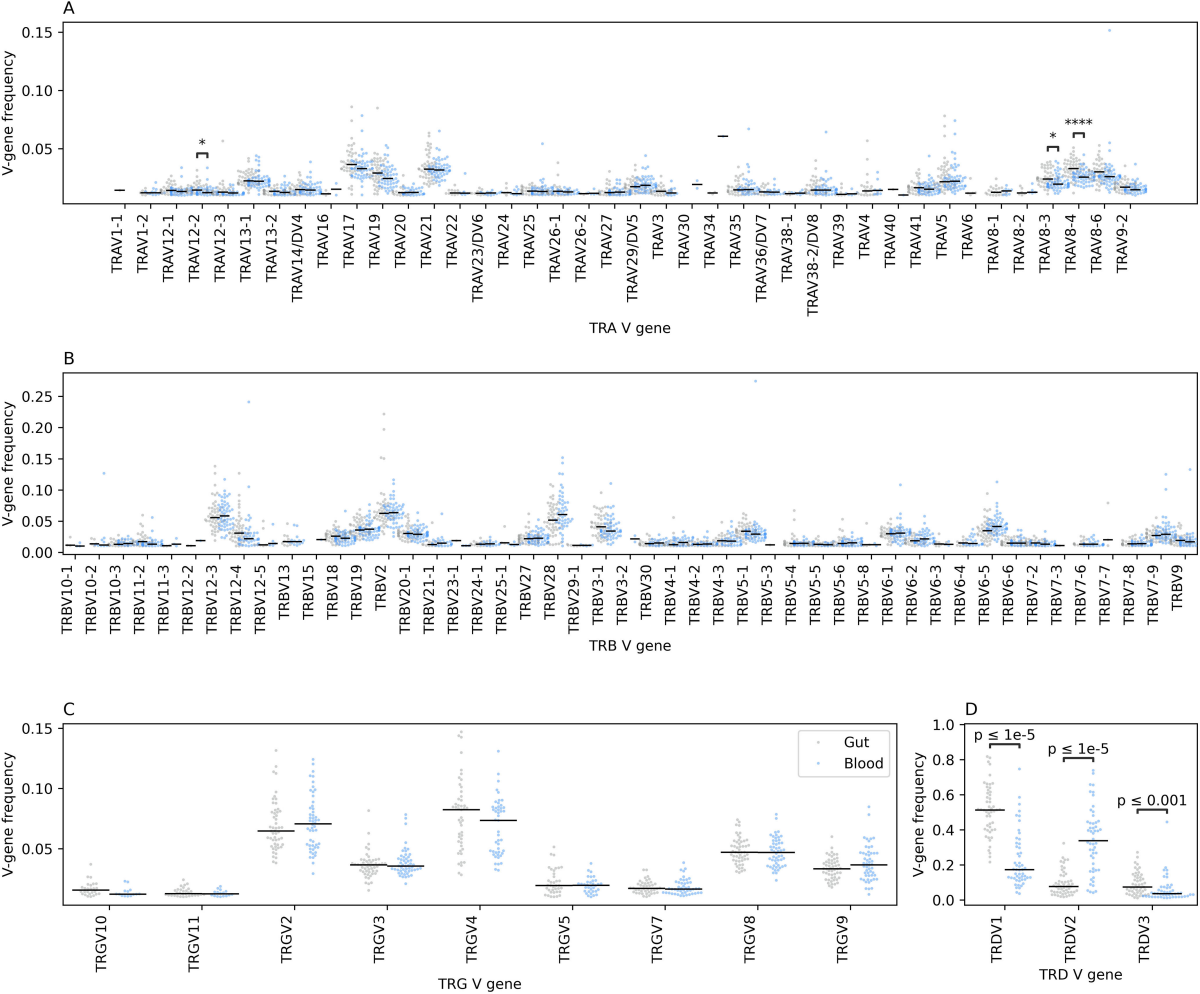
The relative frequency of different V genes among the four TCR chains in the gut and the blood immune repertoires. **(A)** depicts the differences at the TCR alpha (TRA) chain, while **(B)** depicts the differences at the TCR beta (TRB) chain, **(C)** at the TCR gamma (TRG) chain and lastly, **(D)** at the TCR delta (TRD) chain. Across all panels, blacklines represent the median, we also used the two-sided Mann-Whitney-Wilcoxon test to compare the frequency of each V-gene segment among the two anatomical compartments with the Bonferroni correction for multiple testing. Additionally, we filtered V-genes with a frequency less than 0.01, i.e. 1%, from the analysis as well as V-gene segments with more than 1% frequency in less than 10 samples from any statistical comparisons. The asterisks are used to represent the P-values, with * represents P ≤ 0.05, and **** designates a P ≤ 0.0001.

Next, we analyzed the V gene usage in the immune repertoire of blood and gut tissues. Across the TRA repertoire we observed a significant difference in gene usage with *TRAV8-3*, *TRAV8-4*, and *TRAV12–2* showing higher frequency in colonic tissues compared to blood (**Figure 3A**). This prominent difference was not observed at the TRB repertoire (**Figure 3B**). These results suggest that within the αβ T cell population, different cellular subsets preferentially reside in these two anatomical compartments. The same findings were observed in the γδ T cell repertoires, specifically, the TRD repertoire, where *TRDV1* and *TRDV3* had a higher frequency in gut tissues relative to blood while *TRDV2* dominated the blood repertoires (**Figures 3C, D**). TRGV9 preferentially pairs with the *TRDV2* to form the γ9δ2 subset which is the main subset of γδ T cells observed in blood. Whereas we did not observe a significant difference in V-gene utilization at the TRB repertoire, we observed a robust and significant difference at the J gene level, where *TRBJ2–1* and *TRBJ2–2* showed a high expansion in colonic tissues while *TRBJ2–6* and *TRBJ2–7* showed a higher level at the blood level (**Supplementary Figure S1**). Similarly, we observed a significantly higher utilization of *TRDJ1* clonotypes in colonic tissues relative to the blood and an increase in the frequency of *TRDJ3* in the blood relative to colonic tissues (**Supplementary Figure S1**).

Across the three B cell receptor chains, namely, IGH, IGK and IGL, we observed significant differences in V-gene usage between the blood and the gut repertoires (**Supplementary Figure S2**). Specifically, within the IGH repertoire, where *IGHV3–7* and *IGHV3–21* were found at significantly higher frequencies in gut tissues compared to blood, while *IGHV1-69, and IGHV3-30–3* were more frequent in blood than in gut tissues (**Supplementary Figure S2A**). Similar findings were observed at the IGL repertoire where some genes such as *IGLV2-14*, *IGLV-23* and *IGLV2–8* showed a significantly higher abundance in gut tissues relative to blood tissues while *IGLV3–1* showed higher frequency in blood relative to gut tissues (**Supplementary Figure S2B**). The IGK also showed similar patterns, where some V-genes such as *IGKV4-1*, *IGKV3-20*, *IGKV-30*, and *IGKV1–5* showed higher abundance in gut tissues relative to blood, while *IGKV1–8* and *IGKV1–33* genes exhibited a higher frequency in the blood repertoire relative to gut repertoires (**Supplementary Figure S2C**). Given that we analyzed the joint repertoires of individuals with CD and controls, these findings suggest a physiological difference between blood and gut repertoires. Thus, it indicates that CD might lead to tissue-specific changes in the immune repertoire, or that disease-induced changes could vary in magnitude between different tissues.

### Crohn’s disease induced multiple changes in the blood TCR repertoire that are not detected in the gut repertoire

**Figure 4:**
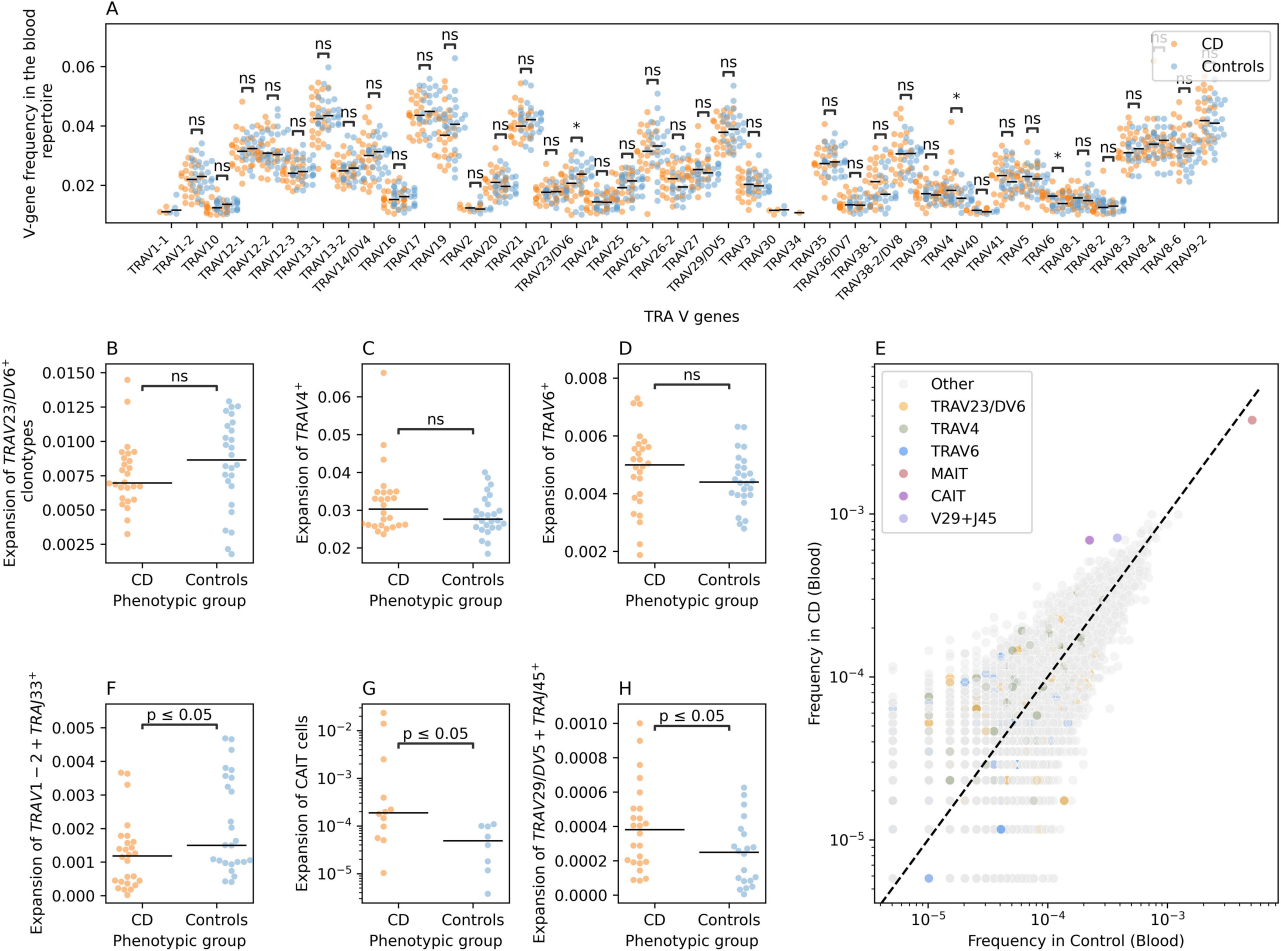
CD-induced changes on the blood TRA repertoire of individuals with CD and controls. **(A)** a comparison between the frequency of different TRA V-genes in individuals with CD and controls. **(B)** shows the difference in the expansion of TRAV23/DV6^+^ clonotypes in CD and controls, while **(C)** illustrates the same relationship but for TRAV4^+^ clonotypes and **(D)** for TRAV6^+^ clonotypes. **(E)** shows the frequency of different VJ-length groups in the TRA blood repertoire of controls and individuals with CD. **(F)** shows the expansion of the TRAV1-2^+^TRAJ33^+^L12^+^ clonotypes which is used as a proxy for MAIT cells in individuals with CD and controls. **(G)** compares the expansion of CAIT cells in individuals with CD and in matching controls. Lastly, **(H)** illustrates the expansion of TRAV29/DV5-TRAJ45+L16 VJ length group in the blood of individuals with CD relative to controls. Across all panels except **(E)**, we used the two-sided Mann-Whitney-Wilcoxon test for statistical comparisons. The asterisks are used to represent the P-values, with n.s. donating non-significant (P>0.05), * represents P ≤ 0.05.

To identify CD induced changes in the blood TCR repertoire, we first compared the frequency of V-genes between individuals with CD and controls (**Figure 4A**). We observed three nominally significant differences in the frequency of three V-genes: *TRAV23/DV6*, *TRAV4*, and *TRAV6* (**Figure 4A**). We then investigated the expansion of clonotypes harboring these V-gene segments in both individuals with CD and controls. Although trends were detected, with a depletion of *TRAV23/DV6* ^+^ clonotypes (**Figure 4B**), and an expansion of *TRAV4* ^+^ (**Figure 4C**) and TRAV6^+^ clonotypes in CD (**Figure 4D**), none of these trends was significant, likely due to the small sample size included in the current study.

To obtain a more detailed view, we categorized clonotypes into refined groups by combining unique V and J genes with specific CDR3 amino acid lengths, creating VJ-CDR3 length groups. This approach revealed several alterations, including a depletion of TRAV1-2+TRAJ33^+^ clonotypes with a CDR3 length of 12 amino acids (TRAV1-2+TRAJ33+L12) in the blood repertoire of individuals with CD (**Figure 4E**). Since the TRAV1-2+TRAJ33 rearrangement is commonly observed in MAIT cells, we labeled this arrangement as MAIT cells. We observed a significant decrease in the frequency of MAIT cells (defined as: TRAV1-2+TRAJ33+L12) in the blood of individuals with CD relative to the blood of symptomatic controls, consistent with our previous findings and those of others (2, 20) (**Figure 4F**).

We also observed an expansion of two other VJ-CDR3 length groups in individuals with CD compared to controls, namely, TRAV12-1+TRAJ6+L15 and TRAV29/DV5+TRAJ45+L16 (**Figure 4E**). The TRAV12-1+TRAJ6+L15 group includes a subset of unconventional T cells we previously identified as Crohn’s-associated invariant T cells (CAIT cells) (2). CAIT cells are characterized by a semi-invariant TCR-alpha chain comprising *TRAV12–1* and *TRAJ6* combination and a CDR3 amino acid sequence represented by the motif CVV**A*GGSYIPTF, where the asterisks represents any amino acids (2). Hence, we compared the expansion of CAIT cells among individuals with CD and controls where we observed a significant expansion in the abundance of CAIT cells in individuals with CD relative to controls, despite our sample size, which corroborated previous findings and suggest that CAIT cells are a key player in the pathogenesis of CD (**Figure 4G**). Lastly, we observed a significant expansion of TRAV29/DV5+TRAJ45+L16 clonotypes in the blood of individuals with CD (**Figure 4H**). To the best of our knowledge, the expansion of this group of clonotypes in the blood of individuals with CD has not been previously reported.

Interestingly, the alterations observed in the blood TRA repertoires were not detected in the gut TRA repertoires (**Supplementary Figure S3**). For example, while comparing the V-gene usage, we observed a nominally significant reduction in the frequency of *TRAV12-1^+^* clonotypes and an increase in TRAV8-6^+^ clonotypes in the blood of individuals with CD relative to controls (**Supplementary Figure S3A**). Nonetheless, we did not detect significant changes in the expansion or depletion of these clonotypes in the gut repertoire of individuals with CD and controls (**Supplementary Figures S3B, C**). Furthermore, candidate clonotype groups identified in the blood repertoire, namely, TRAV1-2+TRAJ33+L12, TRAV12-1+TRAJ6+L15 and TRAV29/DV5+TRAJ45+L16, did not show a differential degree of expansion in individuals with CD relative to controls at the gut repertoire (**Supplementary Figures S3D-3F**), respectively. Additionally, by comparing the frequency of different VJ-CDR3 length groups, we could not identify a group that was significantly different between individuals with CD and controls.

Despite the small sample-size, CD-associated changes were identified in the TRA repertoire, but these did not translate to other TCR chains. For example, we could not detect any significant changes at the TRB repertoire neither in blood nor at the gut level (**Supplementary Figure S4**). Similarly, we failed to detect any significant differences in the γδ T cell repertoires of blood and gut tissues (**Supplementary Figure S5**).

### Crohn’s disease is associated with changes in the IGH repertoire

**Figure 5:**
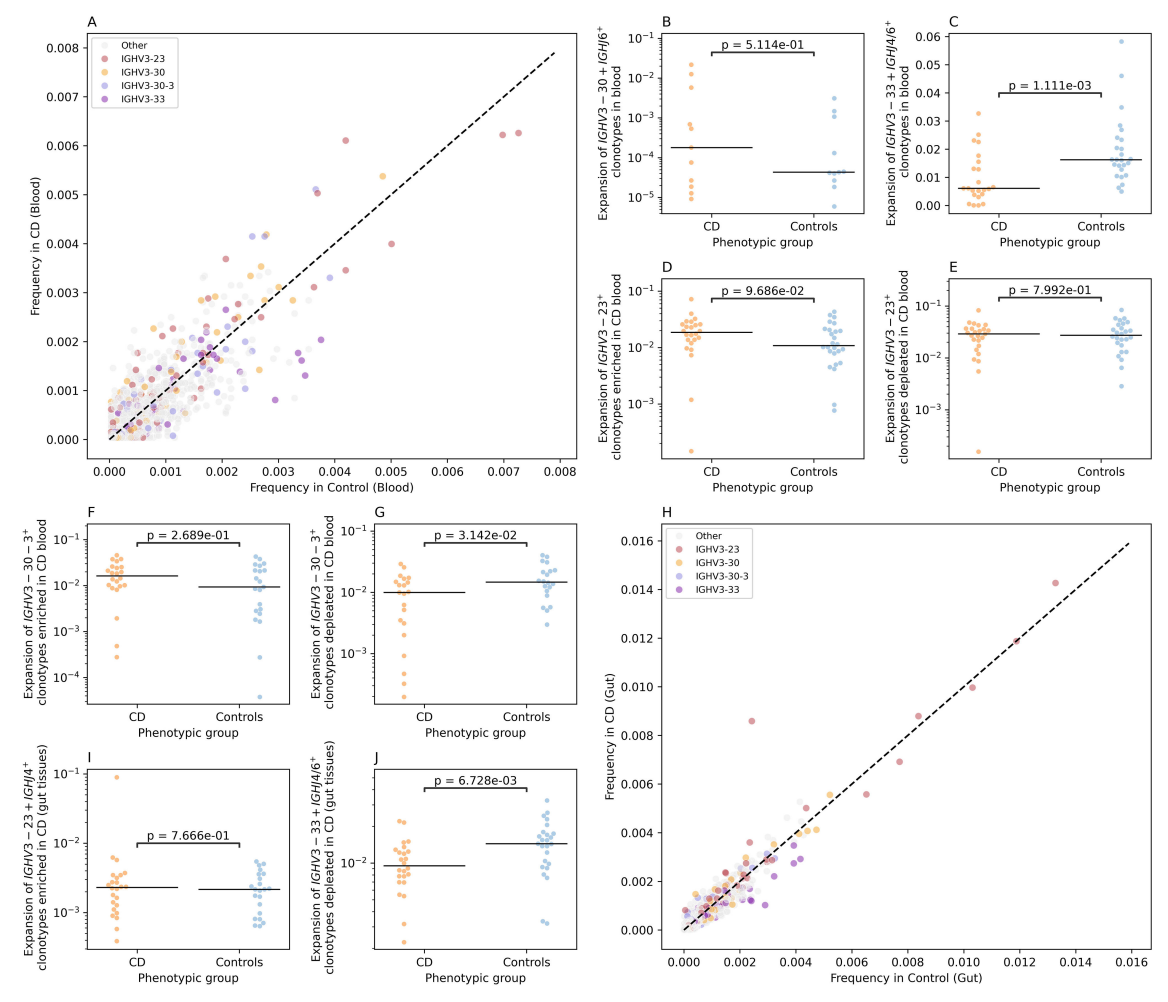
CD-associated changes in the IGH repertoires of colonic mucosa and peripheral blood. **(A)** the differential frequency of VJ-length groups in the blood repertoire of individuals with CD and controls. **(B)** shows the expansion of IGHV3-30+IGHJ6+L24 clonotypes in the blood repertoire of individuals with CD and controls, while **(C)** illustrate the depletion of IGHV3-33+IGHJ4+L18, IGHV3-33+IGHJ4+L19, IGHV3-33+IGHJ4+L16, IGHV3-33+IGHJ6+L25, and IGHV3-33+IGHJ4+L15 clonotypes in the blood repertoire of individuals with CD. **(D, E)** shows the expansion of IGHV3-23^+^ with a frequency higher than 0.002 in the blood of CD and controls, with **(D)** depicting the expansion for VJ-length groups with a higher frequency in CD and **(E)** showing the expansion of VJ-length group with higher frequency in controls relative to CD. **(F, G)** depict the expansion of IGHV3-30–3 VJ-length groups with a frequency higher than 0.002 in the blood of CD and controls with **(F)** showing the expansion of IGHV3-30-3^+^ clonotypes with a higher frequency in CD relative to controls and **(G)** depicting the difference in the expansion of VJ length groups with higher frequency in controls. **(H)** the differential frequency of VJ-length groups in the Gut repertoire of individuals with CD and controls. **(I)** the expansion of IGHV3-23+IGHJ4+L22 clonotypes in the gut IGH repertoire of CD and controls. Lastly **(J)**, depicts the depletion of IGHV3-33+IGHJ4+L18, IGHV3-33+IGHJ4+L19, IGHV3-33+IGHJ4+L16, IGHV3-33+IGHJ6+L25, and IGHV3-33+IGHJ4+L15 clonotypes in the gut IGH repertoire of individuals with CD.

For BCR repertoire analysis, we examined the IGK and the IGL light chains in both blood and gut tissues but found no significant differences between individuals with CD and controls (**Supplementary Figures S6**-**S9**). Subsequently, we focused on the immunoglobin heavy chain repertoire (IGH). By comparing the IGH V-gene frequency in the blood repertoire of individuals with CD and controls we observed a nominally significant reduction in the frequency of *IGHV3-33* ^+^ in the blood repertoire of individuals with CD relative to controls (**Supplementary Figure S10**). Subsequently, we binned clonotypes into groups using the same VJ-length approach discussed above (**Figure 5A**). This enabled us to identify multiple groups that had differential frequency in individuals with CD relative to controls. One of these groups was IGHV3-30+IGHJ6+L24, which had a higher frequency in cases relative to controls, however, by comparing the expansion of this group between individuals with CD and controls we did not detect any significant difference (**Figure 5B**). We also observed a significant depletion of a subset of *IGHV3-33+IGHJ4* ^+^ and *IGHV3-33+IGHJ6^+^* clonotypes, specifically, IGHV3-33+IGHJ4+L18, IGHV3-33+IGHJ4+L19, IGHV3-33+IGHJ4+L16, IGHV3-33+IGHJ6+L25, and IGHV3-33+IGHJ4+L15 (**Figure 5A**). These clonotypes were significantly depleted in individuals with CD relative to controls (**Figure 5C**). We also observed a group of *IGHV3-23^+^* clonotypes that had higher frequency in CD relative to controls (**Figure 5A**).

To disentangle this further, we selected *IGHV3-23^+^* VJ-length group with a frequency above 0.002 (*i.e.* 0.2%) in cases and controls. After that we compared the expansion of these subgroups in individuals with CD (**Figure 5D**) and in controls (**Figure 5E**), i.e. clonotype groups with a higher frequency in CD relative to controls and vice versa, which showed no significant difference. The same framework was used to analyze the IGHV3-30-3^+^ clonotypes, where VJ-length group with higher frequency in CD relative to controls did not show a significant difference in the expansion (**Figure 5F**). On the other side, IGHV3-30-3^+^ VJ length groups with a higher frequency in control, illustrated a significant depletion in the blood repertoires of individuals with CD relative to controls (**Figure 5G**).

Subsequently, we repeated the VJ length group analysis using the gut IGH repertoire (**Figure 5H**). We observed a higher frequency of a specific VJ-length group, namely, IGHV3-23+IGHJ4+L22 in individuals with CD relative to controls (**Figure 5H**). Nonetheless, by comparing the expansion of this group of clonotypes in cases relative to controls, we did not observe any significant differences with only one CD samples showing a high level of expansion (**Figure 5I**). Lastly, we aimed at investigating the IGHV3–33 depletion signal observed at the blood repertoires (**Figure 5C**) in the gut repertoires. To this end, we compared the expansion of *IGHV3-33+IGHJ4* ^+^ and *IGHV3-33+IGHJ6^+^* clonotypes VJ-length groups defined above in the gut IGH repertoire of individuals with CD and controls which confirmed the depletion of these clonotype groups at the local gut repertoire (**Figure 5J**).

## Discussion

To study Crohn’s disease effects on different immune repertoire compartments, we profiled the T and B cell immune repertoires in blood and colonic mucosa from 27 individuals with CD and 27 age-matched symptomatic controls from the IBSEN III cohort (12). This enabled us to identify physiological differences between the blood and the gut repertoire irrespective of the disease status. In addition to identifying disease-alterations that were observed either at the gut and/or the blood. Before discussing the immunological and clinical implications of our findings, we would like to discuss methodological and technical aspects influencing our findings, namely, cellular composition and spatial differences. Peripheral blood contains a large fraction of T (70%) and B (15%) cells (21), however, theses proportions vary across tissues. Although we used an equal amount of DNA (120ng) for profiling the blood and the gut repertoires, differences in cellular composition between these two tissues, does not equate to equal numbers of T and B cells in the starting material. Additionally, spatial heterogeneity within tissues can impact the results of immune profiling. To minimize this variability, we focused on biopsies from the left-side of the colon, including both inflamed and non-inflamed biopsies from individuals with CD and controls. However, this approach may have introduced heterogeneity in the collected gut biopsies limiting our ability to detect CD-associated changes in the gut repertoire.

A potential solution to address this problem is using fluorescence-activated cell sorting (FACS), which can be used to standardize the number of cells derived from each tissue or even sublocations from a specific organ. Nonetheless, this approach requires access to viable cells from the affected tissue which might not be possible for previously collected and achieved samples. Additionally, FACS-approaches might not be suitable for high throughput applications as they are more time and labor intensive relative to direct repertoire profiling from available tissue biopsies. Hence, the choice between the direct profiling of the repertoire or the sorting of cells and then profiling the repertoire depends on the availability of viable cells from the affected tissues as well as the number of cells and the study question.

Despites these technical aspects, our analysis revealed that changes were primarily observed in the TRA repertoire. While other studies have reported alterations in the TRB (17) and the TRG (22) repertoires, they involved larger sample sizes, suggesting that analyzing the TRA repertoire might be a more sample-efficient method for identifying disease-associated clonotypes. A key finding in our study was the depletion of MAIT cells (defined using its semi-invariant alpha-chain) in the blood of individuals with CD, consistent with previous reports (2, 20, 23–25). Using paired measurements of blood and gut samples from the same individual we showed that this effect is only observed in the blood repertoire and not in the gut repertoire. This indicates that this depletion is not due to the migration of MAIT cells to the gut, as individuals with CD and controls had comparable levels of MAIT cells in their gut tissues.

Beyond the decrease in their abundance, MAIT cells in IBD show a different functional and phenotypic profile that is characterized by a reduction in IFN-γ production upon stimulation, in addition to a higher expression of the apoptotic marker annexin V and the exhaustion marker PD-1 (25). The higher expression of proapoptotic markers, which was also observed by Hiejima and colleagues (20), coupled with the expression of exhaustion markers indicate that in IBD, MAIT cells might be decreased because of an exhaustion-induced apoptosis which is observed with chronic T cell activation (26, 27). A recent study by Yasutomi and colleagues (28) highlighted the pathogenic role of MAIT cells in IBD, showing that MR1 deficient mice have a less severe outcome in an Oxazolone-induced colitis model. Interestingly, the authors also reported that the administration of the MR1 antagonist, isobutyl 6-formyl pterin reduced the colitis severity in the same model as well as decreased the production of cytokines by MAIT cells (28).

Nonetheless, our study lacked functional characterization of these depleted MAIT cell populations, such as their transcriptomic landscape or expressed surface markers. Subsequently, it is hard to draw conclusions about the functional state of these MAIT cells, for example, the subpopulation of MAIT mostly affected by this depletion, *e.g.* MAIT1 or MAIT17 subpopulations. Additionally, the anatomical site of MAIT depletion, *e.g.*, colonic tissue or blood. Hence, future studies shall aim at studying the functional state of MAIT cells in the colon and the blood repertoire of treatment-naive individuals with CD. Dysregulated MAIT responses have been previously identified in multiple immune-mediated diseases (IMIDs) such as rheumatoid arthritis (29), systemic lupus erythematosus (30) and primary sclerosis cholangitis (31). These findings highlight the importance of understanding the contribution of MAIT cells to IMIDs.

The immune repertoire represents a promising source for novel therapies to treat IMIDs such as IBD, for example, a novel therapy for ankylosing spondylitis that depends on depleting T cell clonotypes involved in the disease was recently described by Britanova et al. (32). However, to discover these clonotypes and validate their expansion, larger cohorts are needed. In addition to our work on T cells, we performed an in-depth investigation of the blood and gut B cell repertoire in individuals with CD and symptomatic controls. This study represents, to the best of our knowledge, the first bulk gut B cell repertoire profiling in CD as well as paired profiling of the blood and gut B cell repertoires. Our findings highlight the importance of studying tissue-specific B cell repertoires, given the low level of overlap observed between the blood and the gut B cell repertoires. Although we identified multiple abnormalities in both compartments, the functional implications, *e.g.* the target antigen(s), remain unknown. To address this, integrating BCR-Seq methods with large-scale antigen screening methods such as phage-immunoprecipitation sequencing (PhIP-Seq) (33) could enable the identification of antigenic specificities for disease relevant B cell clonotypes.

Our results demonstrate the feasibility and the potential of simultaneous T and B cell repertoire profiling using DNA from blood and tissues samples. However, the small sample size included in the study limits its statistical power and our ability to detect disease-associated changes with weaker effects. In addition, our DNA-based approach did not allow for the identification of the B cell receptor isotypes, *e.g.* μ, γ, or α, limiting insights into the biological significance of the identified clonotypes. This can be attributed to the genetic architecture of the recombined IGH locus where a long intron separates the V(D)J region from the constant region, which hinders the identification of isotype from DNA based assays. Lastly, our profiling method did not contain unique molecular identifiers, which hinders our ability to quantify clonal expansion, conduct reliable somatic hypermutation analysis as well as to remove technical artifacts introduced by PCR amplifications. Future studies should include larger sample sizes, incorporate unique molecular identifiers in the amplicon design, and utilize RNA, whenever possible, to capture the V(D)J recombination along with the heavy chain isotype for a more comprehensive profiling.

## Data Availability

The datasets presented in this article are not readily available due to data privacy regulations in Norway and our institution. However, data are available upon request, if the aims of the planned analyses are covered by the written informed consent signed by the participants, pending an amendment to the ethical approvals and a material & data transfer agreement between the institutions. Requests to access the datasets should be directed to m.l.hoivik@medisin.uio.no.
