# Supplementary material for: Simultaneous profiling of the blood and gut T and B cell repertoires in Crohn’s disease and symptomatic controls illustrates tissue-specific alterations in the immune repertoire of individuals with Crohn’s disease
Source: Front Immunol. 2025 Sep 5;16:1638522. doi: 10.3389/fimmu.2025.1638522 (PMC12447526; doi:10.3389/fimmu.2025.1638522)
Supplement: Supplementary file 1 [file Table1.docx]

**Supplementary Figures**

**
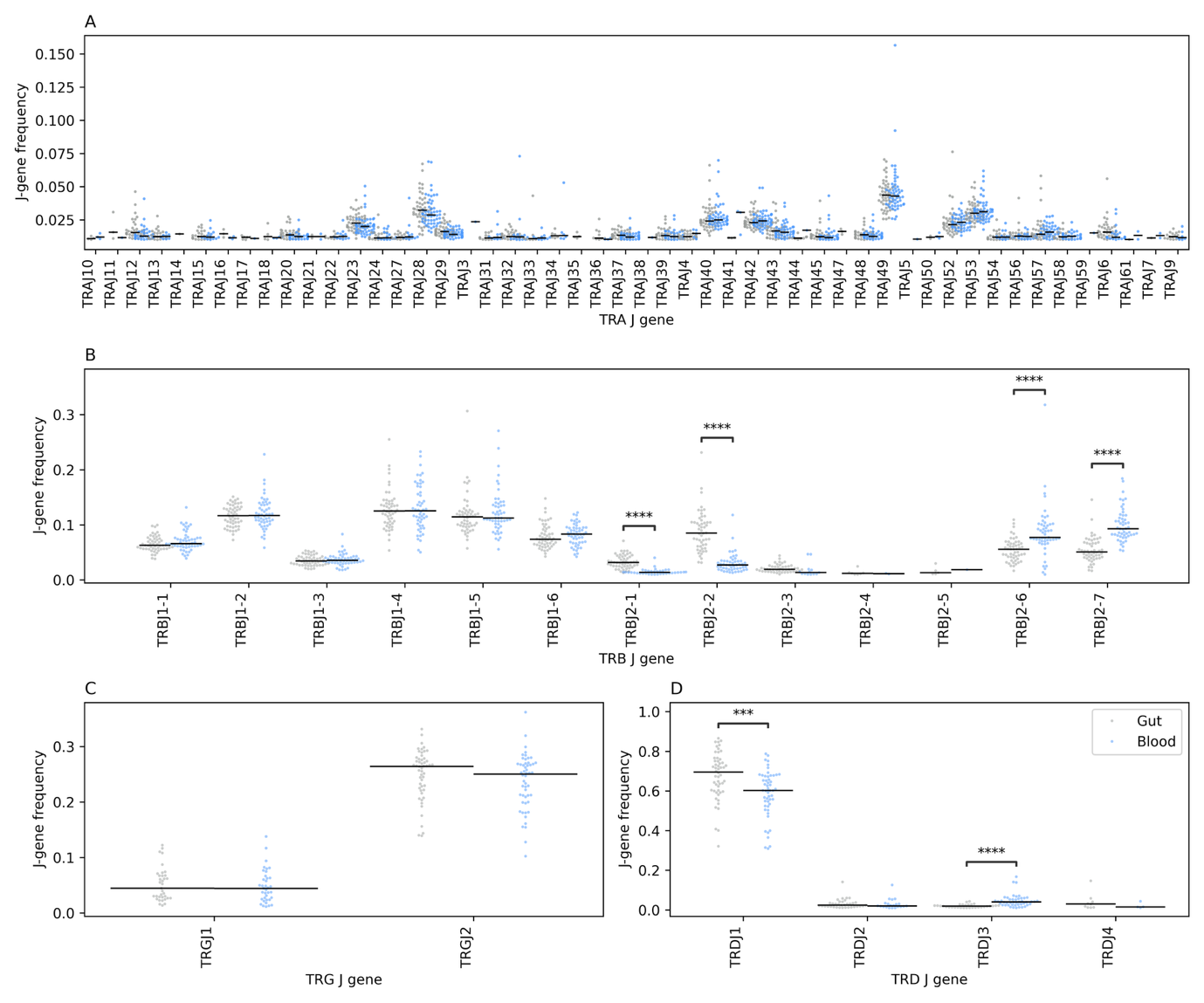
**

**Figure S1:** the relative frequency of different J genes among the four TCR chains in the gut and blood immune repertoires. (**A**) depicts the differences at the TCR alpha (TRA) chain, while (**B**) depicts the differences at the TCR beta (TRB) chain, (**C**) at the TCR gamma (TRG) chain and lastly, (**D**) at the TCR delta (TRD) chain. Across all panels, blackline represents the median, we also used the two-sided Mann-Whitney-Wilcoxon test to compare the frequency of each J-gene segment among the two anatomical compartments with the Bonferroni correction for multiple testing. Additionally, we filtered V-genes with a frequency less than 0.01, i.e. 1%, from the analysis as well as V-gene segments with more than 1% frequency in less than 10 samples from any statistical comparisons.


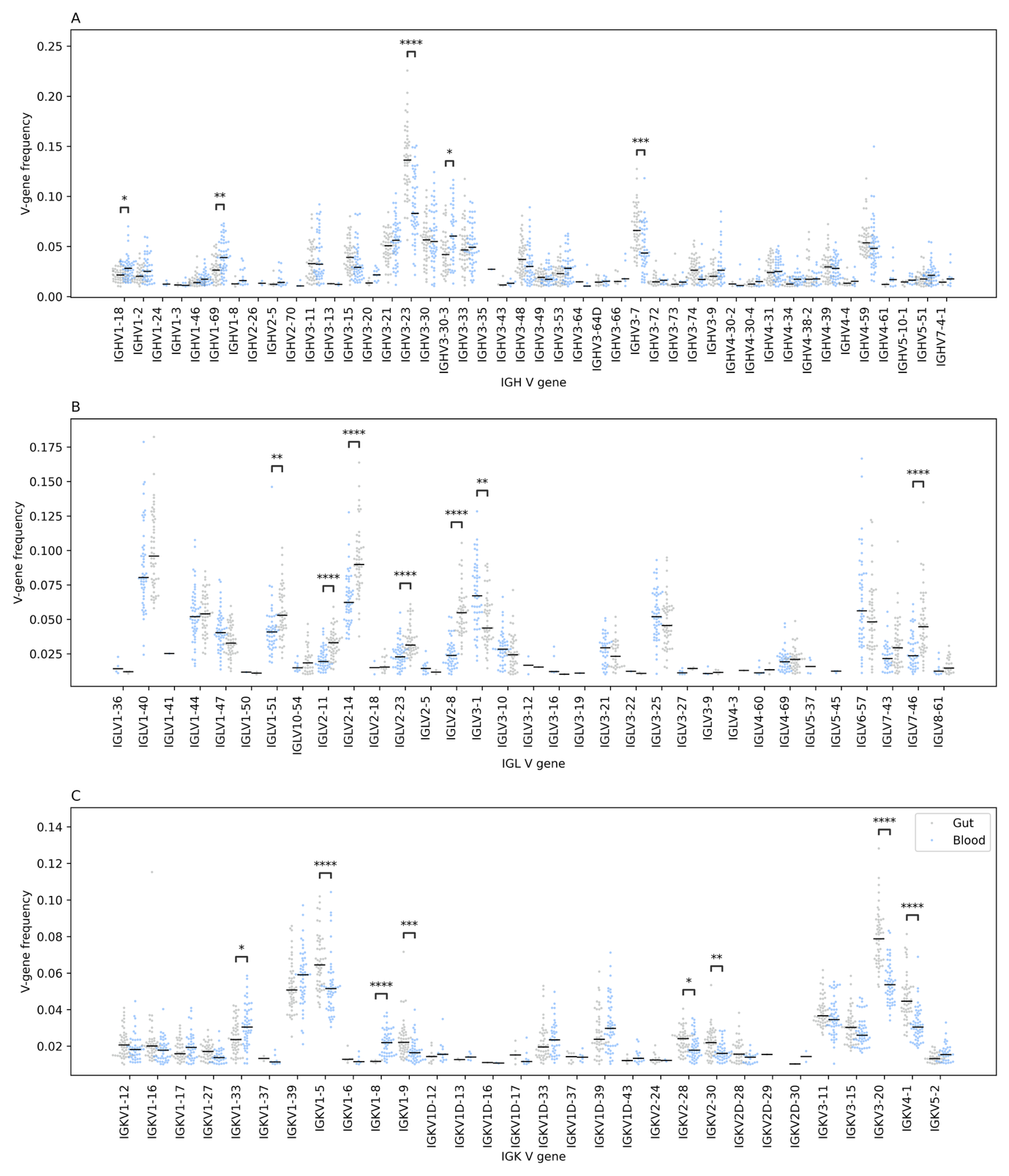


**Figure S2**: the difference in the V gene usage between the blood and gut B cell receptor chain repertoire. (**A**) shows the difference at the immunoglobin heavy chain (IGH), while (**C**) and (**D**) depict the differences in the V-chain usage for the immunoglobin lambda (IGL) and immunoglobin kappa (IGK) chain, respectively. Across all panels, blackline represents the median, we also used the two-sided Mann-Whitney-Wilcoxon test to compare the frequency of each V-gene segment among the two anatomical compartments. Additionally, we filtered V-genes with a frequency less than 0.01, i.e. 1%, from the analysis. Further, we excluded V-gene segments with more than 1% frequency in less than 20 samples from any statistical comparisons.


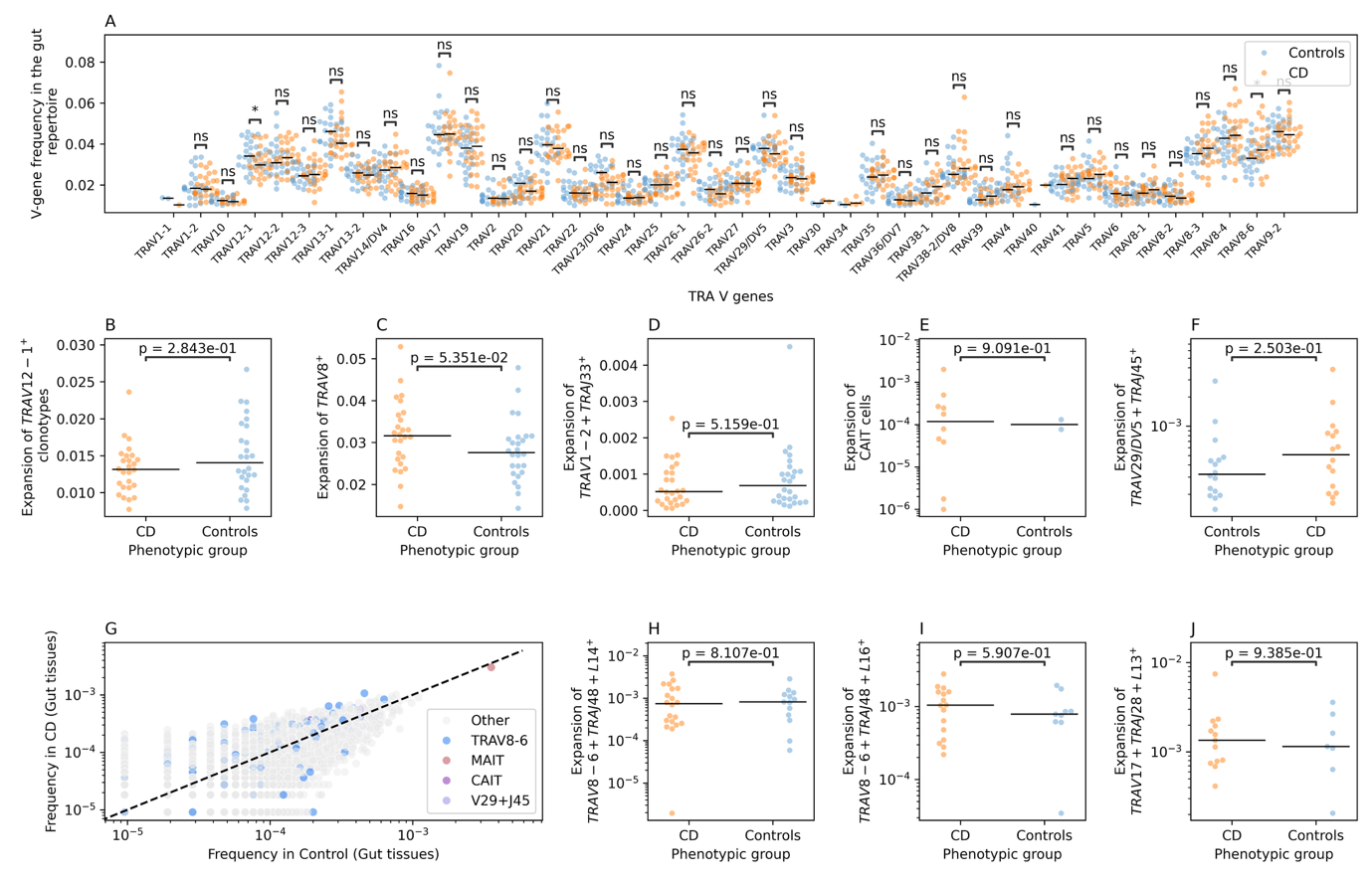


**Figure S3**: CD-induced changes on the gut TRA repertoire of CD patients and controls. (**A**) depicts a comparison between the frequency of different TRA V-genes in CD patients and controls. (**B**) shows the difference in the expansion of TRAV12-1^+^ clonotypes in CD and Controls, while (**C**) illustrates the same relationship but for TRAV8^+^ clonotypes. (**D**) shows the expansion of the TRAV1-2+TRAJ33+L12 which is used as a proxy for MAIT cells in CD patients and Controls, while (**E**) depicts the same relationship but for CAIT cells and lastly, (**F**) illustrate the expansion of TRAV29/DV5-TRAJ45+L16 group in the gut of CD patients relative to controls. (**G**) shows the frequency of different VJ-length groups in the TRA gut repertoire of controls and CD patients. (**H**), (**I**) and (**J**) shows the expansion of three VJ-length groups, namely, TRAV8-6+TRAJ48+L14 (**H**), TRAV8-6+TRAJ53+L16 (**I**), TRAV17+TRAJ28+L13 (**J**) in the gut repertoire of CD patients and controls. Across all panels except (**G**), we used the two-sided Mann-Whitney-Wilcoxon test to compare the frequency or the expansion of different V-gene segments or VJ-length groups between CD patients and controls. In (**A**), we filtered V-genes with a frequency less than 0.01, i.e. 1%, from the analysis and we excluded V-gene segments with more than 1% frequency in less than 10 samples from any statistical comparisons.

**
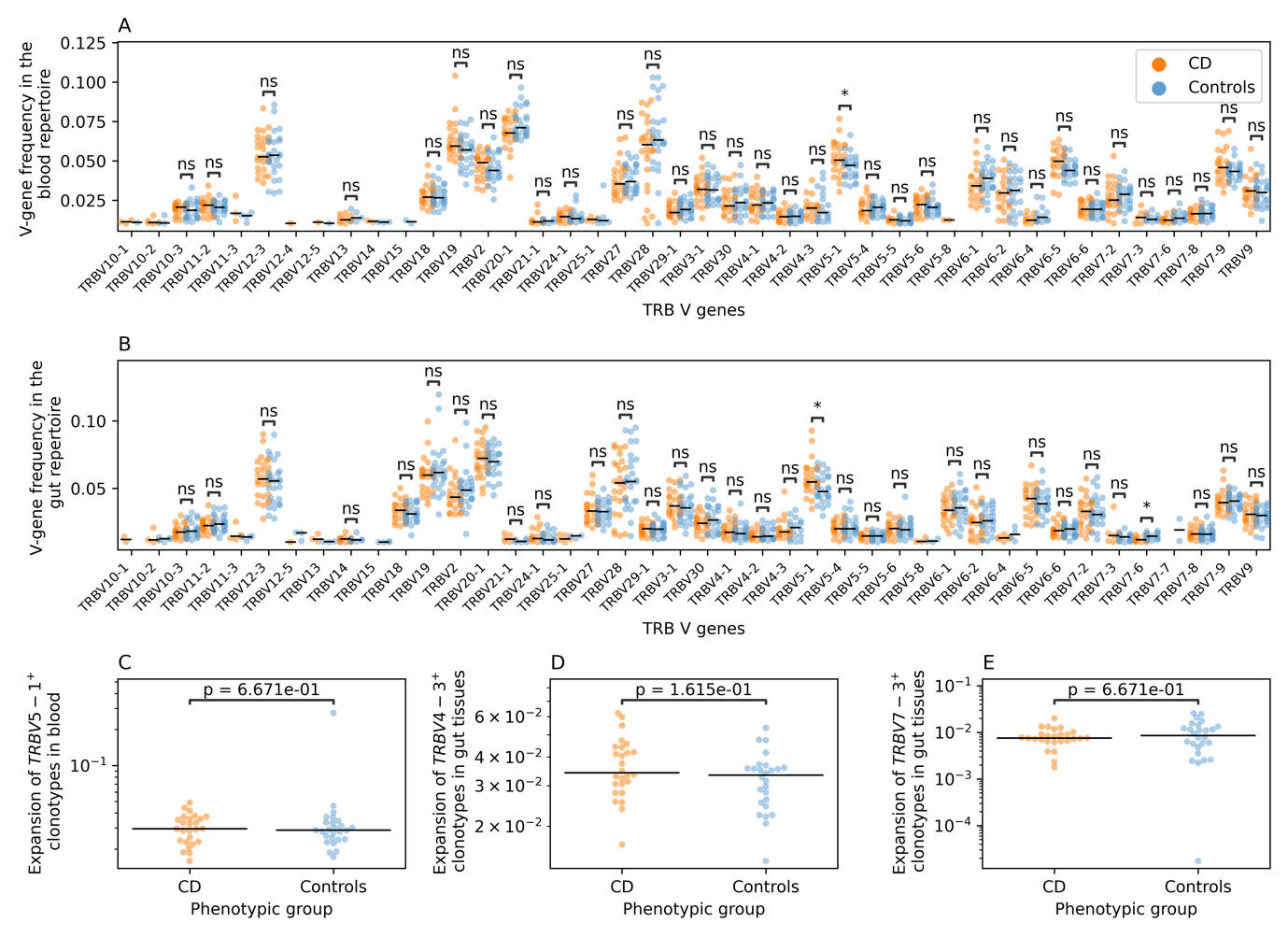
**

**Figure S4:** CD-associated changes in the blood and gut TRB repertoire of CD patients and controls. (**A**) and (**B**) depict a comparison between the frequency of different TRB V-genes in the blood (**A**) and gut (**B**) TRB repertoire of CD patients and controls. (**C**) shows the expansion of TRBV5-1^+^ clonotypes in the blood repertoire of CD and controls while (**D**) and (**E**) show the expansion of TRBV4-3^+^ (**D**) and TRBV7-3^+^ (**E**) clonotypes in the gut repertoire of CD patients and controls. Across all panels, we used the two-sided Mann-Whitney-Wilcoxon test to compare the frequency or the expansion of different V-gene segments or VJ-length groups between CD patients and controls. In (**A**) and (**B**), we filtered V-genes with a frequency less than 0.01, i.e. 1%, from the analysis and we excluded V-gene segments with more than 1% frequency in less than 10 samples from any statistical comparisons.

**
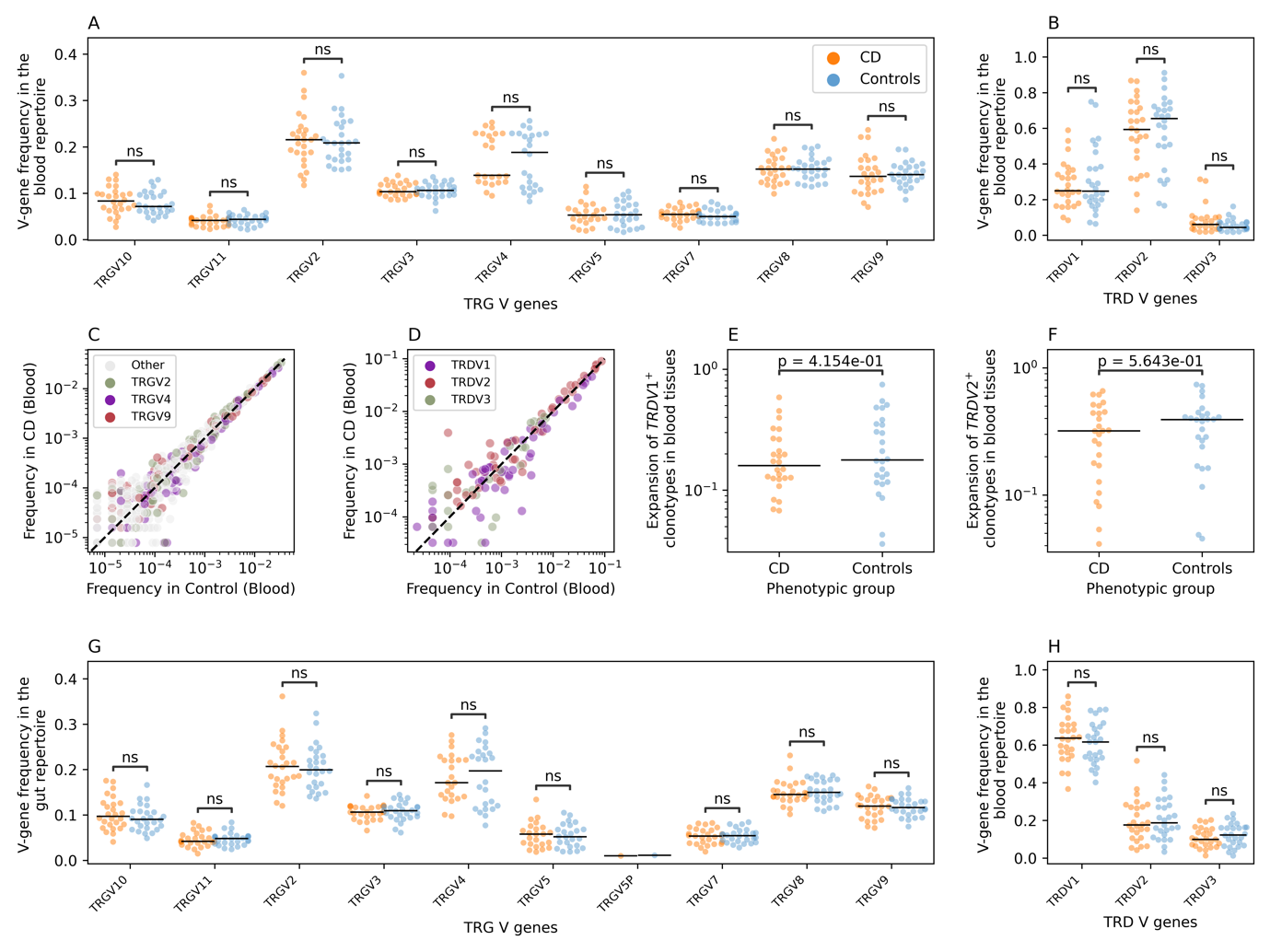
**

**Figure S5:** CD-associated changes at the blood and gut TRG and TRD repertoires of CD patients and controls. (**A**) shows a comparison between the frequency of different TRG V-genes in the TRG blood repertoire of CD patients and controls, while (**B**) depicts the same relation but for the different V genes of the TRD blood repertoire. (**C**) and (**D**) show the frequency of different VJ-length groups in the TRG (**A**) and TRD (**B**) blood repertoire of controls and CD patients. (**E**) and (**F**) shows the expansion of TRDV1^+^ (**E**) and TRDV2^+^(**F**) clonotypes in the TRD blood repertoire of CD patients and controls. (**G**) shows a comparison between the frequency of different TRG V-genes in the TRG gut repertoire of CD patients and controls, while (**H**) depicts the same relation but for the different V genes of the TRD gut repertoire. In (**A**), (**B**), (**G**), and (**H**), we filtered V-genes with a frequency less than 0.01, i.e. 1%, from the analysis and we excluded V-gene segments with more than 1% frequency in less than 10 samples from any statistical comparisons. Across all panels, we used the two-sided Mann-Whitney-Wilcoxon test to compare the frequency or the expansion of different V-gene segments or VJ-length groups between CD patients and controls.


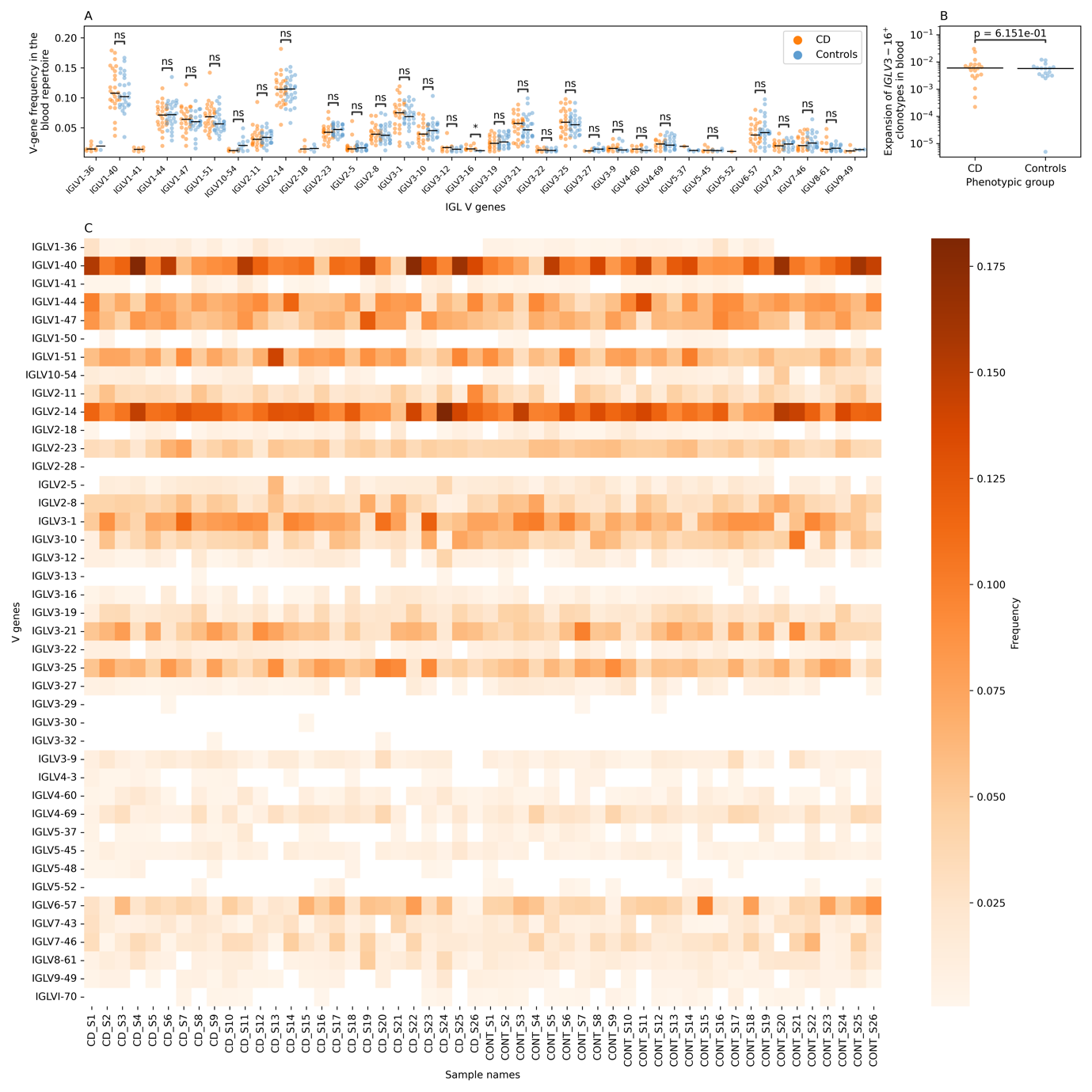


**Figure S6**: CD-induced changes on the IGL blood repertoire of CD patients and controls. (**A**) shows a comparison between the frequency of different IGL V-genes in the IGL blood repertoire of CD patients and controls. In (**A**) we filtered V-genes with a frequency less than 0.01, i.e. 1%, from the analysis and we excluded V-gene segments with more than 1% frequency in less than 10 samples from any statistical comparisons. (**B**) shows the expansion of IGLV3-16^+^ clonotypes in the blood IGL repertoire of CD patients and controls. (**C**) represent a heatmap of the frequency of each V gene in the IGL repertoire of each CD sample (CD_S) and symptomatic control samples (CONT_S) included in the study cohort. In (**A**) and (**B**), we used the two-sided Mann-Whitney-Wilcoxon test to compare the frequency of different V-gene segments (**A**) or the expansion of different VJ-length groups (**B**) between CD patients and controls.

**
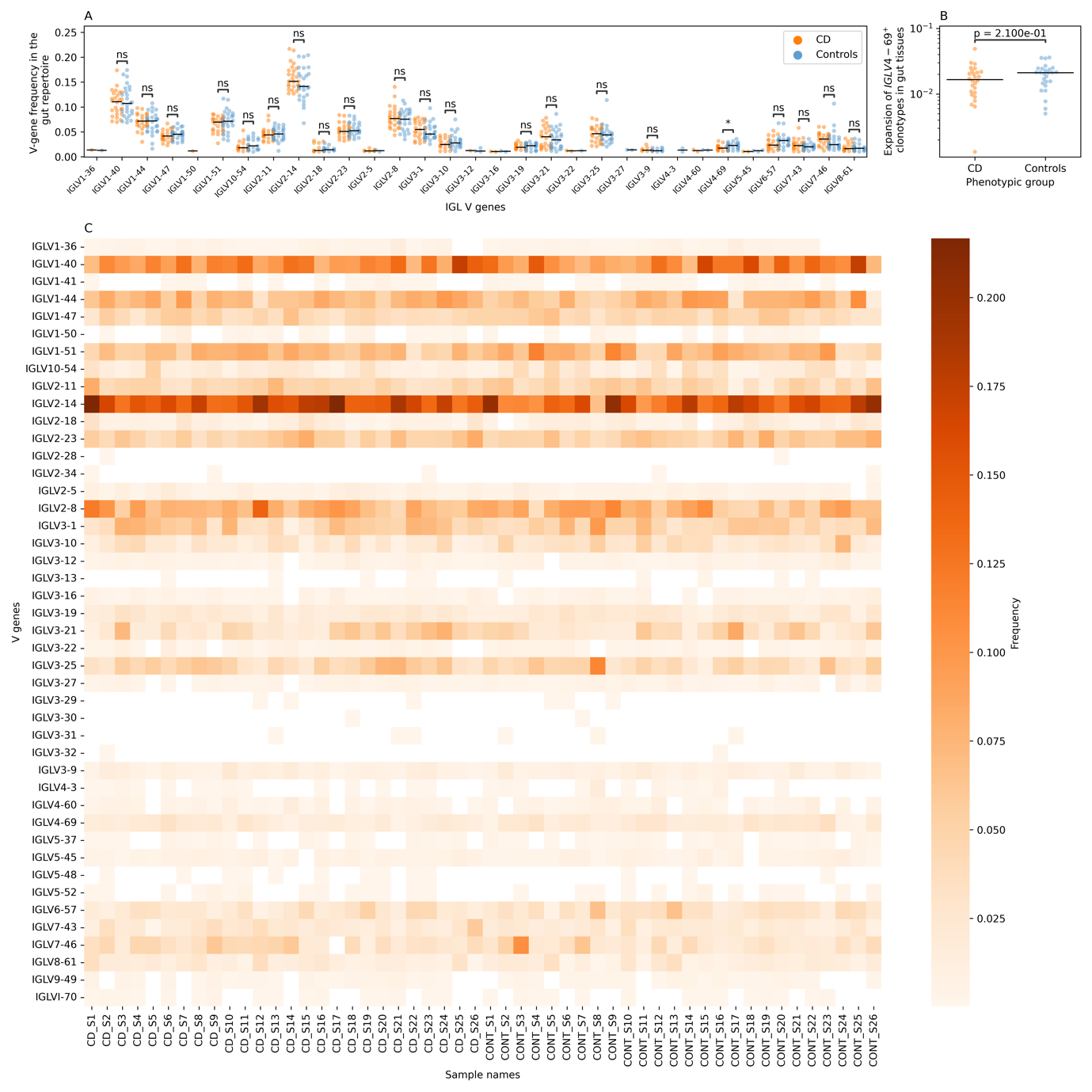
**

**Figure S7**: CD-induced changes on the IGL gut repertoire of CD patients and controls. (**A**) shows a comparison between the frequency of different IGL V-genes in the IGL gut repertoire of CD patients and controls. In (**A**) we filtered V-genes with a frequency less than 0.01, i.e. 1%, from the analysis and we excluded V-gene segments with more than 1% frequency in less than 10 samples from any statistical comparisons. (**B**) shows the expansion of IGLV4-69^+^ clonotypes in the gut IGL repertoire of CD patients and controls. (**C**) represent a heatmap of the frequency of each V gene in the IGL repertoire of each CD sample (CD_S) and symptomatic control samples (CONT_S) included in the study cohort. In (**A**) and (**B**), we used the two-sided Mann-Whitney-Wilcoxon test to compare the frequency of different V-gene segments (**A**) or the expansion of different VJ-length groups (**B**) between CD patients and controls.

**
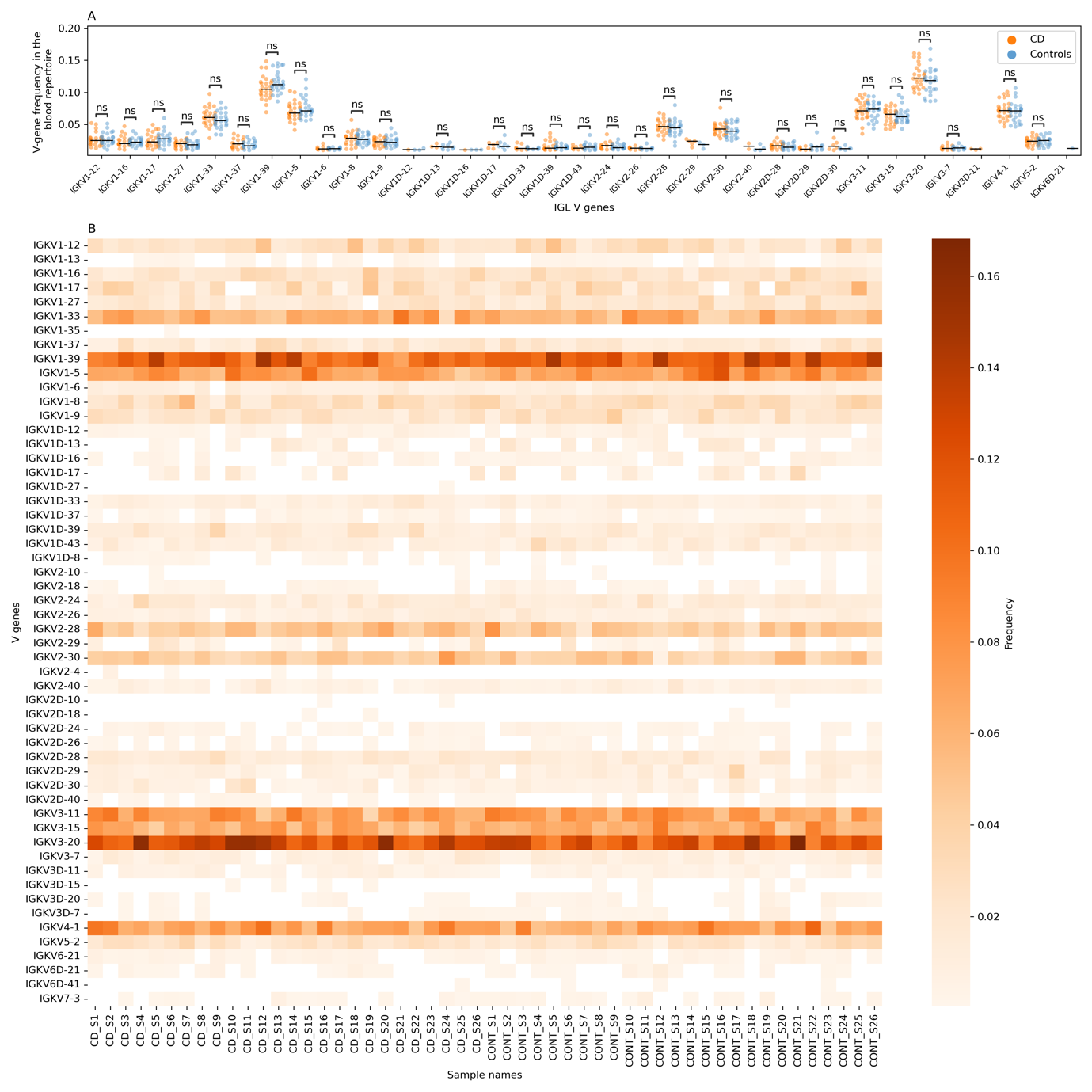
**

**Figure S8**: CD-induced changes on the IGK blood repertoire of CD patients and controls. (**A**) shows a comparison between the frequency of different IGK V-genes in the IGK blood repertoire of CD patients and controls. In (**A**) we filtered V-genes with a frequency less than 0.01, i.e. 1%, from the analysis and we excluded V-gene segments with more than 1% frequency in less than 10 samples from any statistical comparisons. Also, we used the two-sided Mann-Whitney-Wilcoxon test to compare the frequency of each included V-gene segment between CD patients and controls. (**B**) represents a heatmap of the frequency of each V gene in the blood IGK repertoire of each CD sample (CD_S) and symptomatic control samples (CONT_S) included in the study cohort.

**
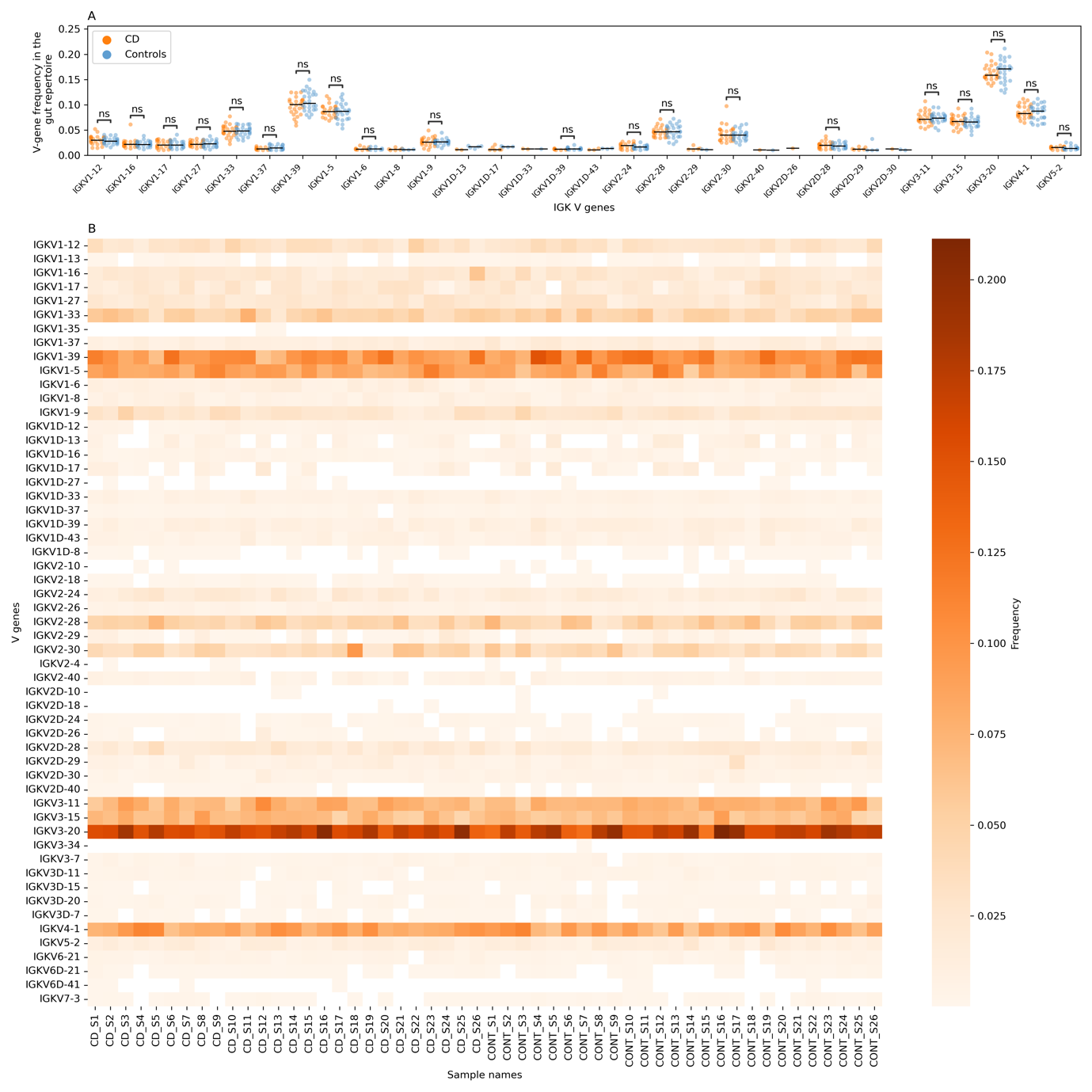
**

**Figure S9:** CD-induced changes on the IGK gut repertoire of CD patients and controls. (**A**) shows a comparison between the frequency of different IGK V-genes in the IGK gut repertoire of CD patients and controls. In (**A**) we filtered V-genes with a frequency less than 0.01, i.e. 1%, from the analysis and we excluded V-gene segments with more than 1% frequency in less than 10 samples from any statistical comparisons. Also, we used the two-sided Mann-Whitney-Wilcoxon test to compare the frequency of each included V-gene segment between CD patients and controls. (**B**) represents a heatmap of the frequency of each V gene in the gut IGK repertoire of each CD sample (CD_S) and symptomatic control samples (CONT_S) included in the study cohort.


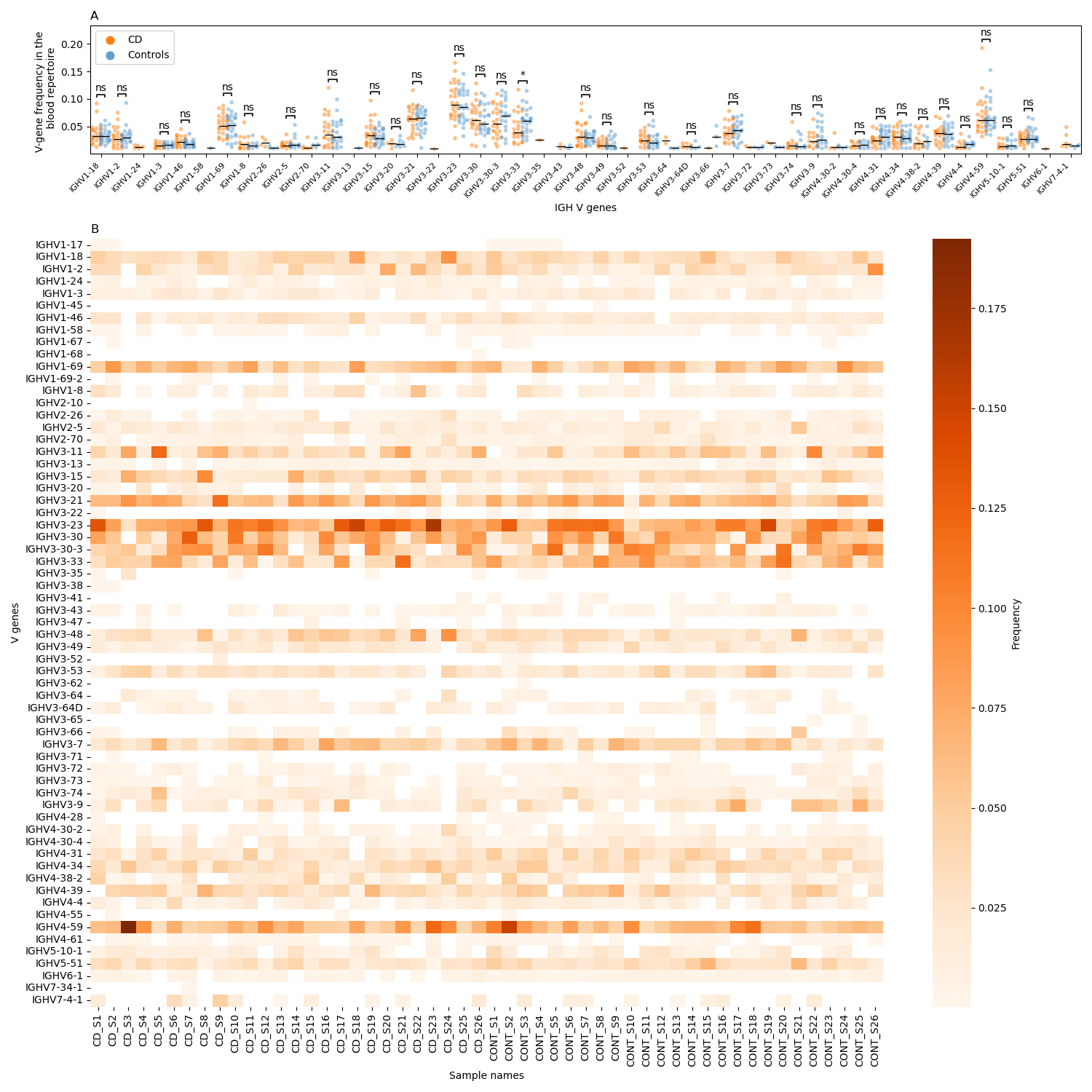


**Figure S10:** CD-induced changes on the IGH blood repertoire of CD patients and controls. (**A**) shows a comparison between the frequency of different IGH V-genes in the IGH blood repertoire of CD patients and controls. In (**A**) we filtered V-genes with a frequency less than 0.01, i.e. 1%, from the analysis and we excluded V-gene segments with more than 1% frequency in less than 10 samples from any statistical comparisons. Also, we used the two-sided Mann-Whitney-Wilcoxon test to compare the frequency of each included V-gene segment between CD patients and controls. (**B**) represents a heatmap of the frequency of each V gene in the blood IGH repertoire of each CD sample (CD_S) and symptomatic control samples (CONT_S) included in the study cohort.
